# A New Method for Common Femoral Arterial Access Using a Mixed Reality–Assisted Technique on a Phantom Model

**DOI:** 10.1177/15266028231208640

**Published:** 2023-11-02

**Authors:** Johannes Hatzl, Daniel Henning, Niklas Hartmann, Dittmar Böckler, Christian Uhl

**Affiliations:** 1 Department of Vascular and Endovascular Surgery, University Hospital Heidelberg, Heidelberg, Germany

**Keywords:** mixed reality, vascular access, endovascular, augmented reality, navigation, vascular surgery

## Abstract

**Purpose:**

The purpose of this study was to investigate the technical feasibility and usability of a mixed reality (MiR)-assisted common femoral arterial (CFA) access technique using a sonography-assisted registration method.

**Materials and Methods:**

A total of 60 CFA punctures were performed on a phantom model by 2 observers. Thirty punctures were performed using MiR (MiR group) and 30 punctures were performed using a conventional sonography-guided access procedure (control group). In the MiR group, a virtual object was created based on a computed tomography (CT) angiography scan of the model and registered to the physical patient in an MiR environment utilizing a software prototype that allowed registration based on a sonography scan. Positional error assessment encompassed 4 measurements using cone beam CT scans: (1) distance of the needle tip to the centerline, (2) distance of the needle entry site from the mid-level of the ostium of the profound femoral artery, (3) angle of entry of the needle in coronal, and (4) sagittal planes. Technical success rates as well as positional errors were compared between both groups. In addition, the usability of the system was assessed according to the system usability scale (SUS).

**Results:**

Technical success was 96.7% and 100% in the MiR and control groups, respectively. The median distance between the needle tip and the centerline was 3.0 (interquartile range [IQR]: 2.0–4.6) in the MiR group and 3.2 mm (IQR: 2.3–3.9) (p=0.63) in the control group. Similarly, the median distance from the needle entry site to the mid-level of the ostium of the profound femoral artery was 3.0 mm (IQR: 2.0–5.0) in the MiR group and 4.5 mm (IQR: 2.0–7.8) (p=0.18) in the control group. The median coronal angles of needle entry were 7.5° (IQR: 6–11) and 6° (IQR: 2–12) (p=0.13), and the median sagittal angles were 50° (IQR: 47–51) and 51° (IQR: 50–55) (p<0.01) in the MiR and control groups, respectively. The mean SUS score provided by both observers was 51.3.

**Conclusion:**

The feasibility of an MiR-assisted CFA access technique could be demonstrated on a phantom model. Further studies are needed to investigate the technique beyond phantom model experiments and in different anatomical settings.

**Clinical Impact:**

This study demonstrates the technical feasibility of a Mixed-Reality-assisted common femoral arterial access procedure on a phantom model. The positional accuracy was comparable to a conventional sonography-guided technique. However, there are several limitations that need to be resolved prior to potential implementation into clinical practice. Further studies are needed to investigate its performance beyond phantom model experiments and the prototypical application requires further technical refinement to increase its usability.

## Introduction

There are several techniques used to perform common femoral arterial (CFA) access for a wide variety of indications. Traditionally, CFA access relies on palpation of the femoral pulse and anatomical landmarks such as the superior iliac spine, the pubic tubercle, and the inguinal crease with or without fluoroscopic visualization of the femoral head. However, the safety of these techniques is compromised due to the inconsistent nature of anatomical landmarks such as the relation of the inguinal crease to the inguinal ligament, especially in unexperienced hands. In addition, anatomical variants such as a high femoral bifurcation or calcifications on the anterior aspect of the vessel are difficult to account for.

Sonography guidance can increase the technical success of CFA access.^1,2^ It offers a 2-dimensional visualization of the target vessel as well as the needle’s tip in real time. Despite its advantages compared with traditional approaches, there are still access-related complications originating from suboptimal puncture presumably due to insufficient visualization of the target and the needle and their respective relative positions in 2 dimensions. In addition, challenges can arise from adverse anatomical situations such as in obese patients or in severely calcified target vessels. Access-related complications can lead to significant morbidity and even mortality.^3–9^

**Figure 1.**
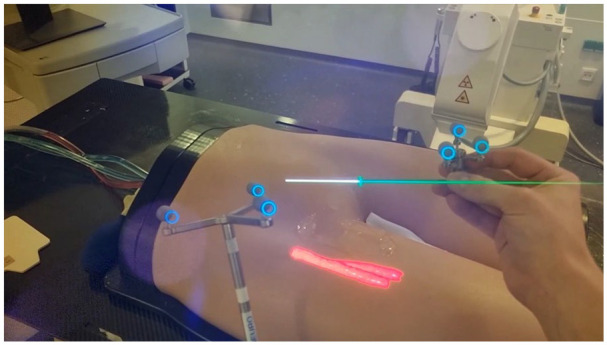
Operator’s point of view performing common femoral arterial access. In this experimental setup the phantom model is placed on an operating table. The Curve Navigation system is used to track the physical and needle positions. A prototypical sonography-assisted registration method was used for the registration of the physical model and the CT-derived 3-dimensional reconstruction of the target vessel. CT, computed tomography.

Mixed reality (MiR) presents an innovate approach, enabling the projection of 3-dimensional virtual objects into the field of view of the observer via a head-mounted display.^10^ The 3-dimensional virtual object can be derived from a computed tomography angiography (CTA) scan. By registering the 3-dimensional virtual object with the physical patient and projecting it directly into the surgeon’s field of view, the operator can use the virtual object to guide the needle into the common femoral artery. Precise registration of the virtual and physical patients is currently the main technical challenge, especially in soft tissue applications. To date, the standard method requires superficial fiducial markers and advanced optical tracking navigation systems. Registration via fiducial markers necessitates the application of these markers on the patient’s skin at the time of the CT scan which severely limits clinical applicability. In addition, slight deviation of the patient’s positioning on the operating table compared with the patient’s position at the time of the CT scan can severely hamper registration accuracy.^11^ In this study, we report a prototypical sonography-assisted registration method (Brainlab AG, Munich, Germany) that uses sonography images to register the physical patient with the virtual CTA-derived imaging. The ultrasound images of the CFA bifurcation that are routinely acquired at the time of the procedure could potentially be used to accurately register the virtual 3-dimensional reconstruction of the patient’s vasculature with the physical patient and subsequently guide the operator (Figure 1, Supplemental Material 1).

The aim of this study was to demonstrate the feasibility of MiR-assisted guidance of CFA access on a phantom model using a prototypical sonography-assisted registration method (Brainlab). In addition, the system’s practical usability from a clinical perspective is reported.

## Materials and Methods

### Phantom Model

**Figure 2.**
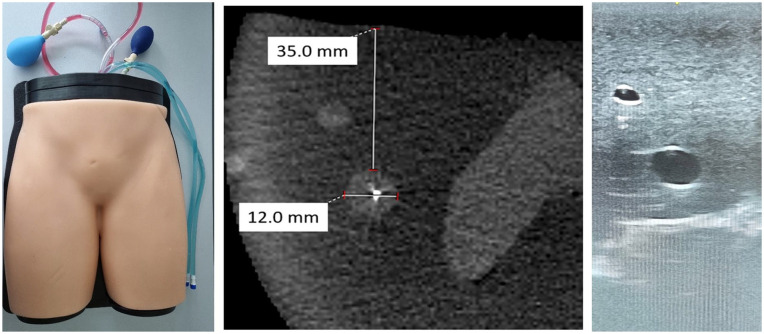
Phantom model with corresponding computed tomography angiography and sonography scans displaying realistic tissue differentiation. The target vessel (CFA) is localized in a depth of 35.0 mm and has a width of 12.0 mm. CFA, common femoral arterial.

**Figure 3.**
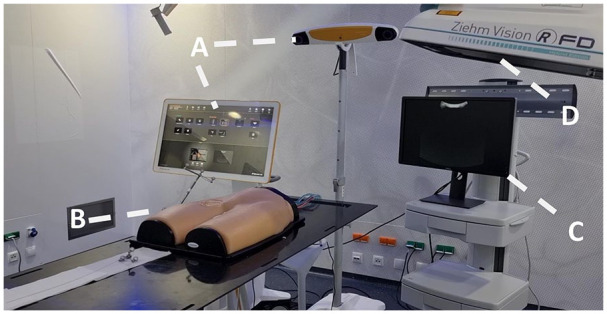
Experimental setup. (A) Curve Navigation platform consisting of the camera and the workstation. (B) Phantom model with a reference array. (C) Screen for sonography-assisted registration. (D) C-arm for cone beam CT scan and radiographic visualization of the guidewire. CT, computed tomography.

**Figure 4.**
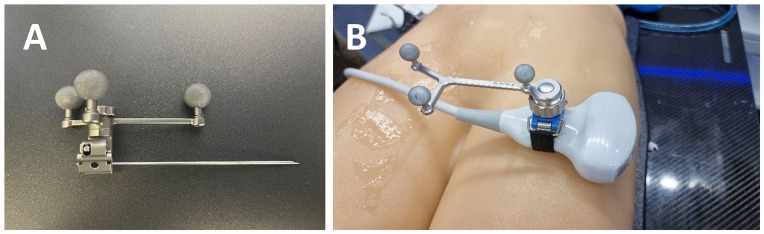
(A) Needle with a reference array. (B) Ultrasound probe with a reference array necessary for sonography-assisted registration.

A Gen II Femoral Vascular Access and Regional Anesthesia Ultrasound Training Model (CAE Healthcare, Mainz, Germany) was used for the purposes of this study. It includes arterial and venous vasculature as well as femoral joints and inguinal ligaments. The model’s soft tissue consists of patented Simulex tissue with self-healing properties that allows 1000+ punctures with minimal residual signs of previous puncture attempts. It can be examined using sonography producing realistic images. The model was filled with radiopaque contrast medium, and a CT scan of the model was performed using an AIRO 32 slice CT scanner demonstrating realistic CT morphological tissue differentiation (Brainlab AG). The target vessel (CFA) is localized in a depth of 35.0 mm and has a width of 12.0 mm. The phantom model with the corresponding CT and sonography images is displayed in Figure 2. The experimental setup is displayed in Figure 3. The required reference tracking arrays necessary for needle tracking as well as sonography-assisted registration are displayed in Figure 4.

### Head-Mounted Display: Magic Leap 2

The Magic Leap 2 has been introduced to the market in September 2022 and has earned IEC 60601 certification which allows its use in the operating room and other clinical settings. It features a 70° field of view and weighs 260 g. Battery life is about 3.5 hours in continuous use.

### Navigation and Registration

For navigation and optical needle tracking, the Curve Navigation platform was used running a modified version of the cranial navigation software (Brainlab AG). For needle tracking by the Curve Navigation system, a reference array was attached to the needle as well (Figure 4). A software prototype was used for sonography-assisted registration of the physical model and the 3-dimensional reconstruction of the CT scan. The software prototype furthermore allowed the visualization of the registered 3-dimensional object as well as the optical tracking of the needle in the MiR environment in the operator’s field of view.

### Workflow: MiR-Assisted CFA Access

A CT scan of the model was performed. The femoral arterial bifurcation was segmented and a virtual 3-dimensional object was created using Mixed Reality Viewer software (Brainlab AG). Segmentation of the femoral bifurcation took about 1 to 3 minutes. The phantom model was positioned on the operating table. The operator was wearing the head-mounted display (HMD), initiated the software, and performed a sonography swipe of the target region using a sonography probe with a tracking array attached to it. The array is necessary to track the positioning of the probe by the Curve Navigation platform. The software algorithm detects the relevant structures within the acquired sonography images that are corresponding with the previous segmentation of the 3-dimensional object from the CT scan and thereby registers virtual and physical objects. By use of an optical marker that is presented to the HMD as well as the Curve Navigation cameras, the registration is subsequently displayed in the field of the view of the operator. The process of registration required about 2 minutes.

Following registration, the operators aimed to puncture the common femoral artery right at the mid-level of the profound femoral artery ostium at a 45° angle and in plane with the vessel’s centerline. The registration was performed individually prior to each attempt. A 14 gauge needle was used and an 0.018″ guidewire was inserted into the vessel. Following every puncture, a cone beam CT scan was performed to visualize the physical needle position using a Siemens Cios Alpha C-arm (Siemens Healthcare GmbH, Erlangen, Germany) (Figure 3). All CFA access procedures in the MiR and control groups were performed in an alternating order between observers. First, the MiR-assisted punctures were performed. The procedures of the control group were performed with a time difference of >4 weeks later. Both observers were familiar with the phantom model and its anatomy and had previously performed CFA access procedures.

Conventional sonography guidance in the control group was performed using an out-of-plane puncture technique.

### Definitions

The precision of the puncture was assessed clinically by inserting a 0.018″ guidewire (Glidewire Advantage, Terumo, Tokyo, Japan). The procedure was defined as technically successful if the guidewire could be inserted into the vessel lumen and visualized radiographically. In addition, a cone beam CTA scan was performed to visualize the actual needle position using centerline reconstructions (3mensio, Pie Medical Imaging BV, Maastricht, the Netherlands). The positional error was then quantified in 4 defined measurements: (1) distance of the centerline to the needle tip in the axial plane, (2) distance of the needle entry point from the mid-level of the profound femoral artery ostium on the centerline, (3) insertion angle in a coronal plane, and (4) insertion angle in a sagittal plane.

In addition, the systems usability was assessed by both operators using the system usability scale.^12^

### Statistical Analysis

Measurements were tested for statistically significant differences using the Welch 2-sample *t* test. Statistical analysis was performed using “R.”^13^ Statistical significance was defined as α<0.05. The system usability score was calculated based on the system usability scale and normalized to 100 as a maximum score. This requires subtracting 1 point from each odd-numbered question, and each even-numbered question’s value was subtracted from 5. The score was added up and multiplied by 2.5. A score of 100 indicates high usability.^14^

## Results

Overall, 60 CFA access procedures were performed on the right CFA of the phantom model. Thirty procedures were performed using the MiR-assisted technique (MiR group). Thirty procedures were performed using the conventional sonography-assisted technique (control group). All procedures were performed by 2 operators, each of whom performed 30 procedures (15 using the MiR-assisted technique and 15 sonography-assisted attempts).

### Technical Success

The guidewire was successfully inserted in 59 of the 60 attempts (98.3%). In 1 case in the MiR group, guidewire insertion was not possible because the needle tip was not adequately positioned within the vessel lumen. Thereby, technical success was achieved in 29 of the 30 (96.7%) in the MiR group and 30 of the 30 (100%) in the control group.

### Positional Errors

**Figure 5.**
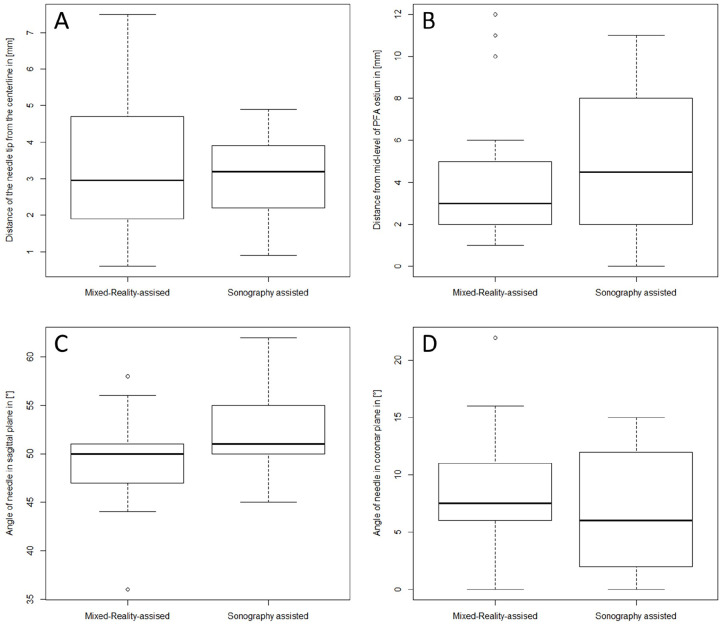
Positional errors: (A) Distance of the centerline to the needle tip in the axial plane. (B) Distance of the needle entry point from the mid-level of the profound femoral artery (PFA) ostium on the centerline. (C) Insertion angle in a sagittal plane. (D) Insertion angle in a coronal plane.

The distance of the needle tip to the centerline was 3.0 mm (interquartile range [IQR]: 2.0–4.6) in the MiR group and 3.2 mm (IQR: 2.2–3.9) in the control group (p=0.63). The distance of the needle entry site from the mid-level of the profound femoral artery ostium was 3.0 mm (IQR: 2.0–5.0) in the MiR group and 4.5 (IQR: 2.0–7.8) in the control group (p=0.18). The angle of the needle in the coronal plane was 7.5° (IQR: 6–11) and 6° (IQR: 2.3–12) (p=0.13) and the angle of the needle in the sagittal plane was slightly but significantly flatter with 50° (IQR: 47–51) and 51° (50–55) in the MiR group and in the sonography-assisted group, respectively. Positional errors are presented in Table 1 and Figure 5.

**Table 1.** Measurements of Positional Errors.

	Mixed reality–assisted	Sonography-assisted	p value^a^
Distance of the needle tip to the centerline (mm)	3.0 (IQR: 2.0–4.6)	3.2 (IQR: 2.2–3.9)	0.63
Distance of the needle entry site from the mid-level of the PFA ostium (mm)	3.0 (IQR: 2.0–5.0)	4.5 (IQR: 2.0–7.8)	0.18
Angle of the needle in the sagittal plane (°)	50 (IQR: 47–51)	51 (IQR: 50–55)	0.005
Angle of the needle in the coronal plane (°)	7.5 (IQR: 6–11)	6 (IQR: 2.3–12)	0.13

Medians with IQR.

Abbreviations: IQR, interquartile range; PFA, profound femoral artery.

a Welch 2-sample *t* test.

### System Usability

Both operators agreed or strongly agreed that the system was unnecessarily complex, although the support of a technical person was not required. Both operators also agreed that the system had too much inconsistency in its current version. Nonetheless, both raters agreed that they would like to use the system frequently. The total system usability score was 51.3. Assessment of the system’s usability is presented in Table 2.

**Table 2.** Mean Results of Usability Rating of Both Observers Using the System Usability Score.

	Strongly disagree								Strongly agree
	1		2		3		4		5
1. I think that I would like to use this system frequently								X	
2. I found the system unnecessarily complex						X			
3. I thought the system was easy to use			X						
4. I think that I would need the support of a technical person to be able to use this system		X							
5. I found the various functions in this system were well integrated			X						
6. I thought there was too much inconsistency in this system								X	
7. I would imagine that most people would learn to use this system very quickly				X					
8. I found the system very cumbersome to use				X					
9. I felt very confident using the system					X				
10. I needed to learn a lot of things before I could get going with this system			X						

## Discussion

This study demonstrates the technical feasibility of MiR-assisted CFA access in a phantom model utilizing a sonography-assisted registration method. Thereby, the need for superficial fiducial markers could be eliminated, which constitutes a main limitation of previously described MiR applications on soft tissues and is 1 important step toward a clinically feasible workflow in the future.^11,15–17^

The use of sonography guidance can reduce the complication rate from CFA access to 1% to 3%.^2,18^ However, due to the large absolute number of patients undergoing CFA access worldwide for a variety of indications, this rate is of clinical relevance. Mixed reality might offer an alternative to further increase the safety of the procedure.

Previous studies on the application of MiR in the field of vascular medicine relied on “paired-point-registration” or manual registration methods with varying degrees of complexity ranging from steering an electromagnetically tracked catheter through a phantom model to the implantation of a scalloped endovascular graft in porcine aortas. A case report of a peroneal arterial access procedure using MiR has also been described in the literature.^11,15,17,19^

In contrast to “paired-point-registration” with fiducial, superficial markers, the sonography-assisted registration method described in this study enables the operator to easily repeat registration and thereby makes it less prone to errors resulting from changes of the physical patient position.

In this experimental setup, the 3-dimensional virtual CFA bifurcation was successfully registered with the physical phantom model with subsequent simultaneous optical tracking of the needle allowing a spatial and 3-dimensional perception of the target structures as well as the needle path in real time directly in the operator’s field of view utilizing a latest generation HMD.

There was 1 technical failure in the MiR group, presumably due to accumulation of registration errors. Otherwise, the technical success rate and positional errors using MiR-assisted CFA access were generally comparable with the technique of sonography-guidance in the present experiment. Furthermore, it was shown that MiR access procedures were performed in a slightly flatter sagittal angle closer to the targeted angle of 45°. However, the difference was very small and of questionable relevance considering this experimental setup.

There are several limitations of this study that need to be considered. First, this is a phantom model study and results might not be generalizable to clinical routine use. Second, although all procedures were performed by 2 observers and in an alternating order, there was only 1 phantom model anatomy in use. Therefore, results with regard to accuracy might also be not generalizable due to learning curve effects. Third, the phantom model had straightforward anatomy. The performance of the technique in adverse anatomical situations such as obesity or severe calcification in comparison with sonography-guided CFA access will need to be investigated in the future.

Despite the elimination of superficial markers, the infrastructural requirements of the method in its current version as well as associated costs were still high, mainly due to the necessity of an advanced optical tracking navigation system that is necessary for instrument tracking (Curve Navigation platform, Brainlab AG). Furthermore, usability scoring of the workflow revealed that this prototypical application requires further adaptation before it can advance to a robust clinical tool. Some steps of the workflow such as needle calibration could be eliminated completely in the future by providing a standardized needle with known dimensions. Continuous development of the sonography registration algorithm as well as sonography per se might improve the technology’s clinical applicability and extend its use beyond access procedures and toward more complex endovascular use cases. The combination of 3-dimensional visualization of anatomy and electromagnetic instrument tracking in situ might harbor the potential to significantly reduce radiation exposure and simplify complex procedures in the future.^17^

## Conclusion

The feasibility of an MiR-assisted CFA access technique could be demonstrated with promising technical success on a phantom model. Further studies are needed to investigate the technique’s performance beyond phantom model experiments and in different anatomical settings. In addition, the usability assessment of this prototype application highlights the need for its continued development.
